# Gene-centric coverage of the human liver transcriptome: QPCR, Illumina, and Oxford Nanopore RNA-Seq

**DOI:** 10.3389/fmolb.2022.944639

**Published:** 2022-12-05

**Authors:** Ekaterina V. Ilgisonis, Elena A. Ponomarenko, Svetlana N. Tarbeeva, Andrey V. Lisitsa, Victor G. Zgoda, Sergey P. Radko, Alexander I. Archakov

**Affiliations:** Institute of Biomedical Chemistry, Moscow, Russia

**Keywords:** transcriptomics, human genome, qPCR, Illumina RNA-Seq, Oxford Nanopore Technologies MinION

## Abstract

It has been shown that the best coverage of the HepG2 cell line transcriptome encoded by genes of a single chromosome, chromosome 18, is achieved by a combination of two sequencing platforms, Illumina RNA-Seq and Oxford Nanopore Technologies (ONT), using cut-off levels of FPKM > 0 and TPM > 0, respectively. In this study, we investigated the extent to which the combination of these transcriptomic analysis methods makes it possible to achieve a high coverage of the transcriptome encoded by the genes of other human chromosomes. A comparative analysis of transcriptome coverage for various types of biological material was carried out, and the HepG2 cell line transcriptome was compared with the transcriptome of liver tissue cells. In addition, the contribution of variability in the coverage of expressed genes in human transcriptomes to the creation of a draft human transcriptome was evaluated. For human liver tissues, ONT makes an extremely insignificant contribution to the overall coverage of the transcriptome. Thus, to ensure maximum coverage of the liver tissue transcriptome, it is sufficient to apply only one technology: Illumina RNA-Seq (FPKM > 0).

## Introduction

The Human Genome Project, which was successfully completed at the turn of the 2nd millennium, gave rise to the technology for analyzing the genomes of living systems. The initial dominance of sequencers relying on polymerase chain reaction (PCR) to prepare sequencing libraries was followed by the creation of a completely new nanopore technology for reading genomes and transcriptomes without using PCR (Jain et al., 2016). Moreover, nanopore sequencing allows for direct reading of RNA chains. Until now, both sequencing technologies have dominated life science-related research, since they allow both reading the sequence of nucleic acids and measuring the content of individual molecules in a biological material, acting in a manner similar to a Geiger counter when measuring ionizing particles (Friedman and Birks, 1948; Ilgisonis et al., 2021).

At the same time, progress with regard to transcriptomic analysis has not been impressive. It remains unclear what could become the gold standard in transcriptomics (Ilgisonis et al., 2021). The analysis of transcripts using the latest generation sequencers is currently based on the use of reads (or fragments) per kilobase per million parameters (Bullard et al., 2010), which is the number of mRNA fragments normalized in the corresponding analytical systems. However, this approach allows only for the relative characterization of the transcript of a particular biological object (Zhao et al., 2020).

The sensitivity of technologies is actually a function of the cut-off lines; for example, for transcriptomic RNA-Seq technology, it is the RPKM (or FPKM) value. At high RPKM/FPKM values, the sensitivity of technologies drops sharply (Łabaj and Kreil, 2016). Thus, the gold standard for characterizing the transcriptome of this biological object remains to be established.

To address this aspect, we applied a well-known chromosome-centric approach that prevails in genomics and analyzed the dependence of the sensitivity of transcriptomic analysis on the RPKM parameter, in relation to the analysis of gene products located on the same chromosome (Ilgisonis et al., 2021). To investigate the dependence of the completeness of coverage of chromosome 18 (Chr18) in the human liver and HepG2 cells, it was concluded that with high sensitivity (when the RPKM values are close to 0), transcriptome sequencing may cover >90% of the entire genome encoded by Chr18 (Ilgisonis et al., 2021).

Here, we do not aim to discuss the possibility of false-positive results, since the final result is known in advance; this is the complete Chr18 genome. By changing the RPKM value, which is the sensitivity of transcriptomic analysis, we analyzed for the most complete coverage of the genome of this chromosome, even in differentiated organs, such as the liver. It can be argued that with a low RPKM value, it is possible to achieve almost complete coverage of the chromosome genome combining three technologies: Illumina RNA-Seq, Oxford Nanopore Technologies (ONT), and quantitative polymerase chain reaction (qPCR) analysis. The best results were achieved using Illumina RNA-Seq.

In this study, we describe our approach in detail; that is, analyzing the averaged results as previously reported (Ilgisonis et al., 2021) and exploring the individual liver by analyzing each liver transcriptome from three donors, using the three technologies mentioned. Our findings suggest that sequencing using Illumina RNA-Seq is sufficient to achieve more than 95% of the individual coverage of the human Chr18 genome of the liver tissue in distinct donor samples.

We applied the approach to investigate other human chromosomes, both short and long, using two technologies—Illumina RNA-Seq and ONT. Based on the results of the analysis of Chr18 liver tissue, we concluded that Illumina RNA-Seq alone is enough to detect the maximum number of transcripts in the sample. Sequencing of the HepG2 cell line has previously shown that ONT could significantly improve the results of Illumina RNA-Seq (Ilgisonis et al., 2021).

Our data confirmed that the combination of the two technologies made it possible to achieve a more complete coverage of the chromosome. However, the difference between the overall coverage using the two technologies and that of the same chromosome using Illumina RNA-Seq alone is not always significant. The theoretical limit of transcriptome coverage is approximately 20,000 transcripts, based on the number of protein-coding genes, neglecting the transcripts of proteoforms (Ponomarenko et al., 2016). The bioinformatics approach based on the combined “low cut-off results” of different technologies enabled the detection of almost all transcripts in the human genome.

## Results

The similarity of the datasets was evaluated using a well-understood example of sets of transcripts encoded by the genes of human chromosome 18. We matched the sets of gene names encoding transcripts found in the liver tissue samples by Illumina RNA-Seq (FPKM > 0), qPCR (number of cDNA copies per cell, Сt ≤ 40; Сt is the threshold cycle, i.e., the number of amplification cycles after which the fluorescence of the split probe exceeded the background values), and ONT (TPM > 0). The Tanimoto index (T) was used for the evaluation. It was estimated that T (RNA-Seq and PCR) = 0.75, T (ONT and PCR) = 0.67, and T (RNA-Seq and ONT) = 0.63, indicating that the datasets obtained by RNA-Seq and qPCR technologies can be considered identical, whereas the other combinations (ONT and qPCR) and (RNA-Seq and ONT) showed much weaker similarities.

**FIGURE 1:**
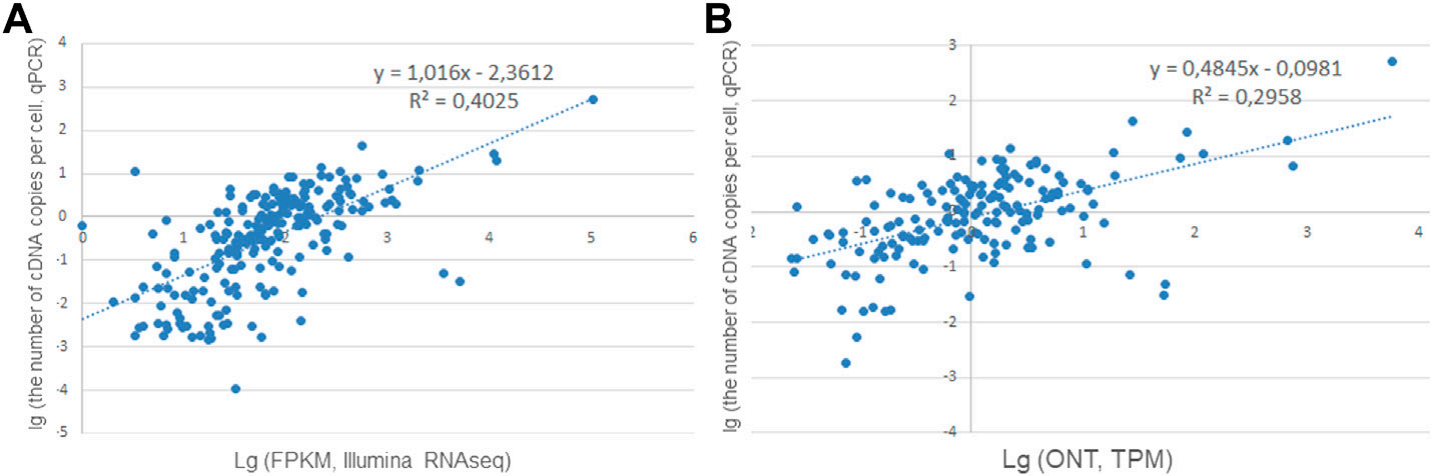
Correlation curves for converting **(A)** Illumina RNA-Seq data (FPKM, *n* = 223) and **(B)** ONT data (TPM, *n* = 171) into the number of cDNA copies per cell (PCR data).

To convert the RPKM (FPKM) and TPM values into the number of copies per cell, calibration curves were constructed using the qPCR data for the results obtained using Illumina RNA-Seq and ONT. The calibration curves and equations are shown in Figure 1.

Comparison of the results of the analysis of the sample taken from one individual showed that transcripts for 223 genes were detected when PCR and Illumina were used simultaneously (the analysis was carried out for genes encoded by chromosome 18), while only 171 genes were detected when PCR and ONT were used, which was 25% less (Figures 1A,B). This decrease could be due to the fact that the sequencing depth for direct RNA sequencing using a single flow cell—the approach employed in our study—is far from the saturation in transcript detection (Soneson et al., 2019).

The relationship between the quantitative results of Illumina RNA-Seq and PCR was stronger than that between the results of ONT and PCR. At the same time, the values *R* ^2^ = 0.4 (Figure 1A) and *R* ^2^ = 0.29 (Figure 1B) do not allow the conversion of FPKM or TRM values (for ONT) into transcripts as copies per cell (without splicing) by using the resulting regression equation: an acceptable value of the determination coefficient (*R* ^2^) must be greater than 0.5 for the model to convert the averaged RNA-Seq RPKM values into copy numbers.

In our previous study, we considered the average values of the results of transcriptome profiling across three donors (Ilgisonis et al., 2021). The study evaluated the relationship between the RPKM parameter and the Tanimoto index. The Tanimoto index was higher (maximum Tanimoto index = 1 and completely matched data arrays) at low RPKM values. We observed that the lower the RPKM cut-off threshold, the higher the Tanimoto index, and more protein-coding genes of the genome were characterized by detectable transcripts.

**FIGURE 2:**
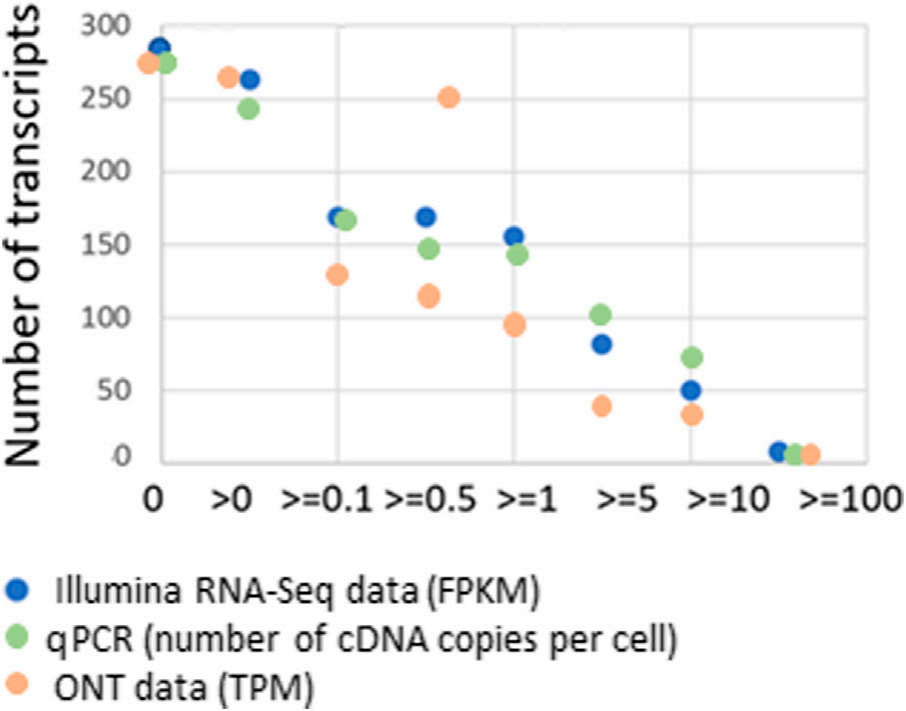
Dependence of the number of detected liver tissue sample transcripts encoded by chromosome 18 (*n* = 275) for various platforms: Illumina RNA-Seq data (FPKM), qPCR (number of cDNA copies per cell, Сt ≤ 40), and ONT data (TPM) on the cut-off level and the concordance of the results obtained with the known genome of chromosome 18.

Figure 2 shows the dependence on the number of detected transcripts. Henceforward, we only describe the “master transcripts,” which represent the primary translation of the coding sequence and resemble at least one of the known isoforms, encoded by the gene (Archakov et al., 2011), given the cut-off level for Illumina RNA-Seq (FPKM), qPCR (number of detected transcripts, Сt ≤ 40), and ONT (TPM).

As the cut-off level increased, the number of detected transcripts increased. Conversely, maximum coverage of the genome by transcripts was achieved at the lowest cut-off level (>0). Thus, with RPKM > 0, transcripts corresponding to 267 genes (97%) were detected, and when moving the cut-off level up to 10 (RPKM > 10), transcripts drastically decreased to 63, only 23% of the total number of Chr18 protein-coding genes. That is possible assuming that we increase the cut-off level to infinity and the number of transcripts becomes zero.

To solve the problem of the most complete transcriptome coverage and, in our case, the maximum number of genes for which the corresponding transcript has been detected, it is advisable to use a minimum cut-off level (i.e., RPKM/FPKM > 0) for the Illumina RNA-Seq data, the number of detected transcripts (Сt ≤ 40) for qPCR data, and TPM > 0 for the results of ONT analysis.

**FIGURE 3:**
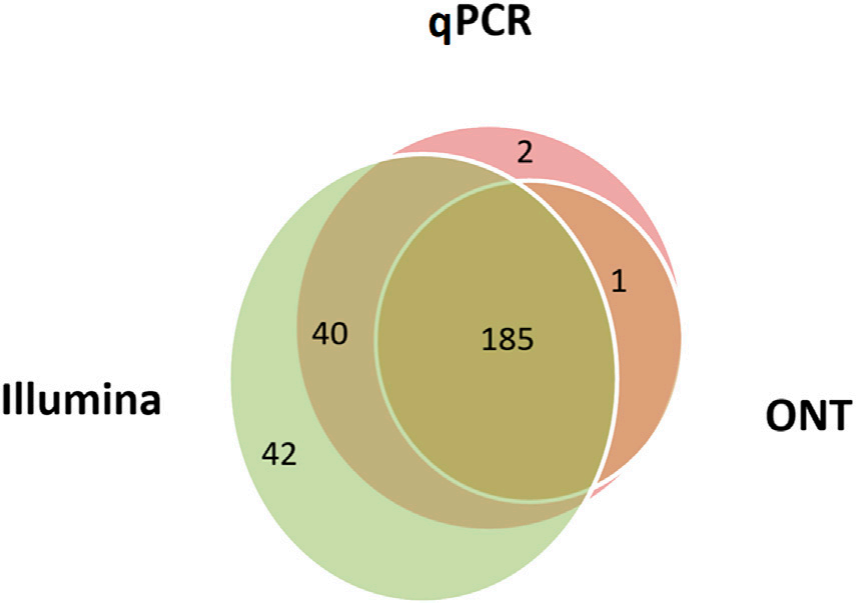
Combined data on the human liver tissue samples indicating the number of chromosome 18 transcripts detected using Illumina RNA-Seq data (FPKM>0), qPCR (the number of cDNA copies per cell, Сt ≤ 40), and ONT data (TPM > 0). A total of 270 transcripts out of 275 encoded on the selected chromosome were detected using all three methods.

By applying the minimum cut-off levels, it was possible to measure the genome coverage for each of the three technologies (Figure 3). The maximum number of transcripts (*n* = 267) was detected using the Illumina RNA-Seq method, and the minimum (*n* = 186) was detected using ONT. As previously mentioned, it is likely that the ONT method in the format used in our study (direct RNA sequencing) has limitations due to a modest sequencing output (1–1.5 gigabase).

### The influence of analytical technology on the assessment of variability in the coverage of expressed genes

**FIGURE 4:**
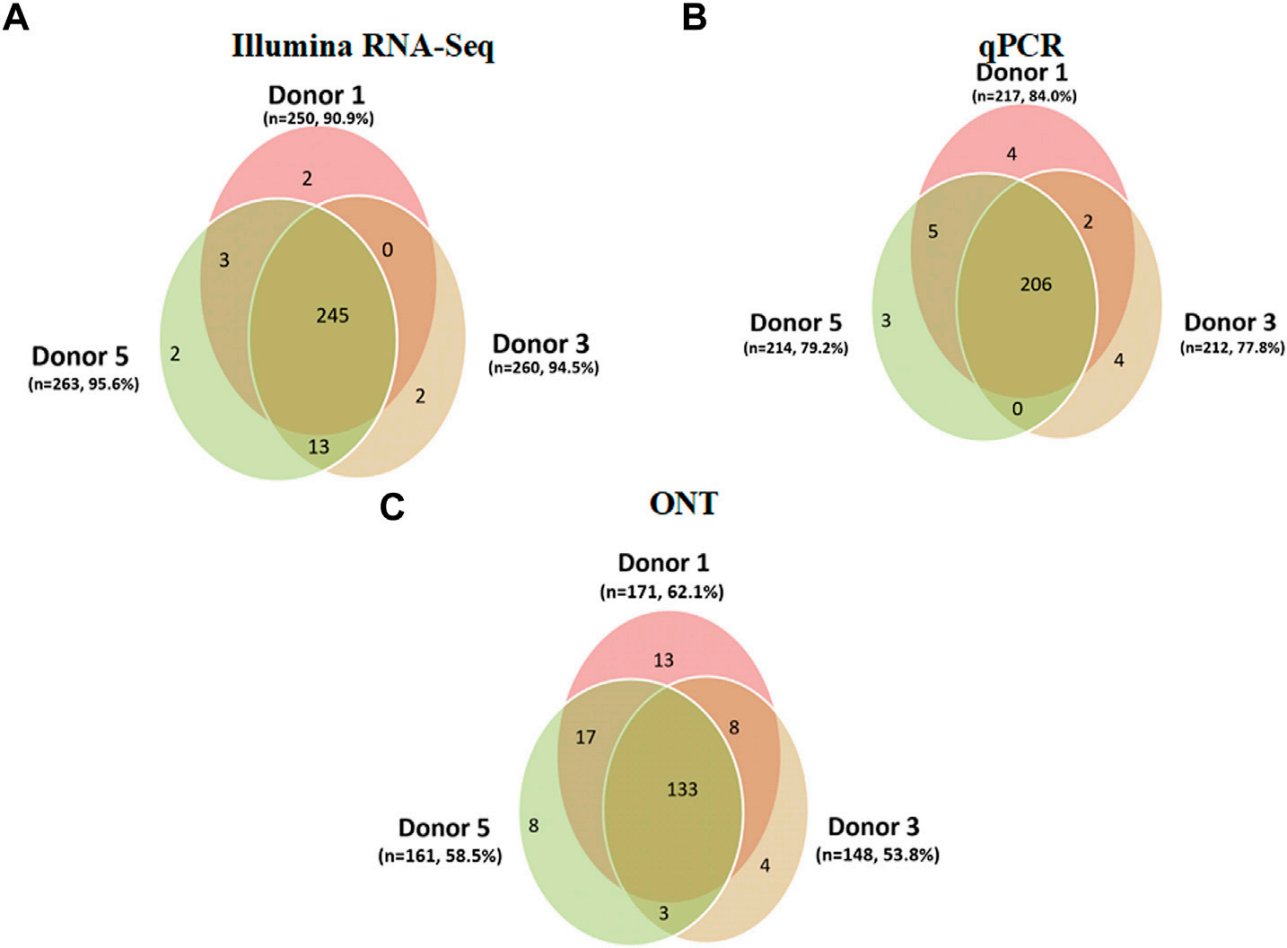
Results of the analysis of the three human liver tissue samples (donor 1, donor 3, and donor 5) indicating the number of the Chr18 transcripts (*n* = 275) detected using **(A)** Illumina RNA-Seq data (FPKM > 0); a total of 267 transcripts were detected, corresponding to 97.0% of the total number of protein-coding genes. **(B)** qPCR (number of cDNA copies per cell, Сt ≤ 40); a total of 224 transcripts were detected (81.4%). **(C)** ONT (TPM > 0); a total of 186 transcripts were detected (67.6%).

In this study, we focused on the assessment of variability in the coverage of expressed genes in the results of transcriptomic profiling of liver tissues from three donors. These donors were three male individuals whose death was not associated with liver damage. The intersection of their transcriptomic profiles is shown in Figure 4. We compared the results obtained for Chr18 genes for each of the three donors using Illumina RNA-Seq (Figure 4A), qPCR (Figure 4B), and ONT (Figure 4C).

The results shown in Figure 4 indicate modest differences between the samples: each of the three technologies deciphered few donor-specific transcripts. These differences ranged from 13 (ONT, donor #1) to 2 (Illumina, donors #1 and #3) transcripts, the genome coverage for Illumina was maximal, and insignificant individual fluctuations were detected. The variability in the coverage of expressed genes of human liver tissue transcriptomes was small, regardless of the analysis method used (Illumina RNA-Seq, qPCR, or ONT). The mean variability was calculated as the average ratio of the number of transcripts specific for each donor divided by the total number of transcripts detected using a certain technology. The values obtained were 0.7% for Illumina RNA-Seq, 1.6% for qPCR, and 4.4% for ONT.

Figure 4 shows the difference between the analytical methods in terms of coverage: the maximum number of transcripts was detected using Illumina RNA-Seq (*n* = 267, Figure 4A) and the minimum was using the ONT method (*n* = 186). Figure 4 shows that in the case of transcriptome profiling of liver tissues, the maximum coverage of the Chr18 genome was achieved using Illumina RNA-Seq. The coverage of each donor was over 90% of the Chr18 genome (90.9%, 94.5%, and 95.6%). The second highest coverage was achieved using qPCR, which made it possible to detect 84%, 77.8%, and 79.2% of the Chr18 genome. The smallest coverage was demonstrated by ONT: 53.8%, 58.5%, and 62.0% (donors #1, #3, and #5, respectively). Interestingly, for all three technologies, the overlap between the results obtained for the three donors was greater than 95%.

**FIGURE 5:**
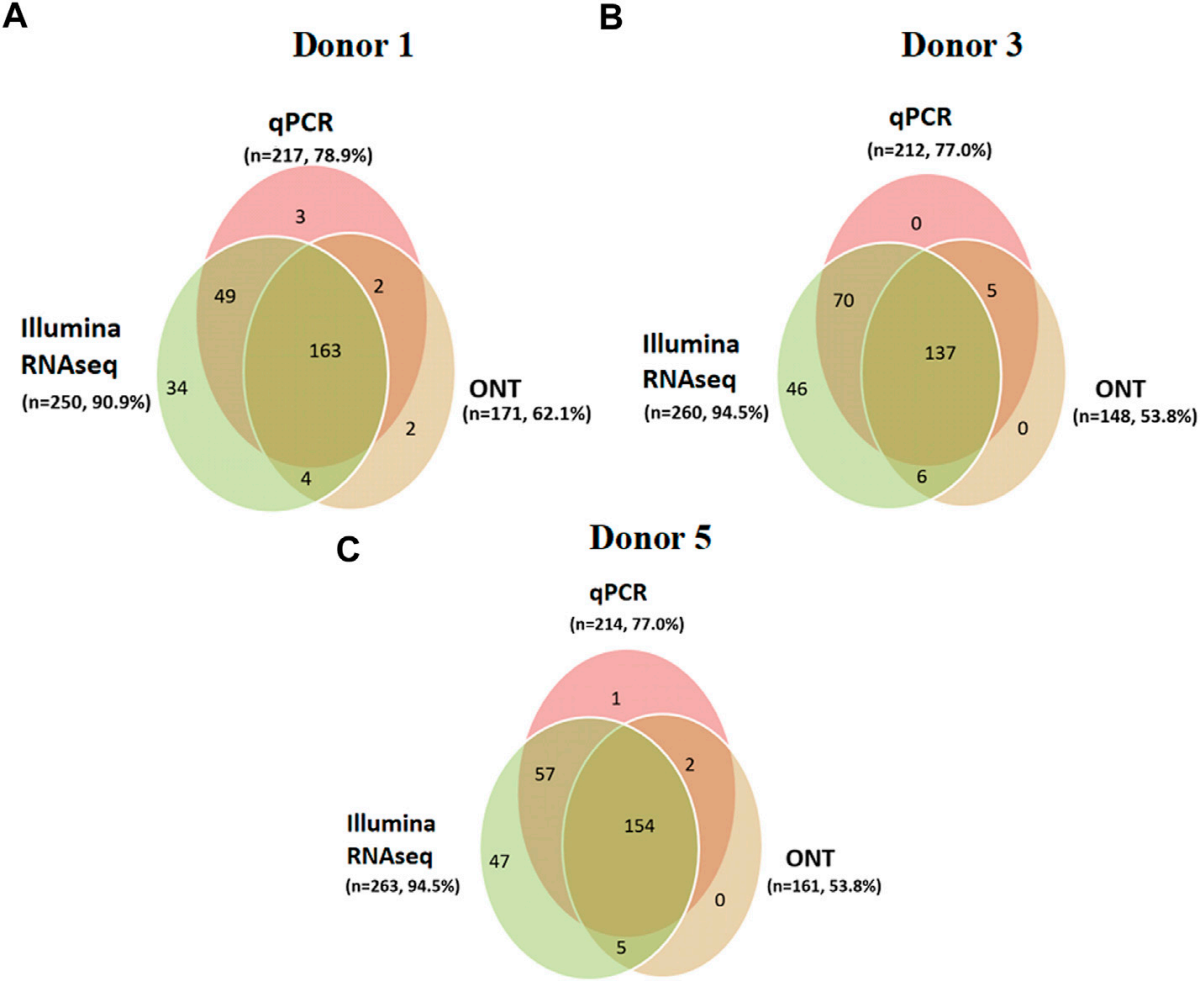
Donor-centric transcriptomes (Illumina RNA-Seq (FPKM > 0), qPCR (number of cDNA copies per cell, Сt ≤ 40), and ONT (TPM > 0)) of human liver tissue samples: **(A)** donor 1 (*n* = 257), **(B)** donor 3 (*n* = 264), and **(C)** donor 5 (*n* = 266), indicating the transcripts encoded by the Chr18 genes (*n* = 275).

Furthermore, the contribution of each method to the coverage of an individual transcriptome was analyzed. For this purpose, intersections between the transcripts detected using Illumina RNA-Seq, PCR, and ONT were evaluated for each donor sample. The results of the analysis are shown in Figure 5.

**FIGURE 6:**
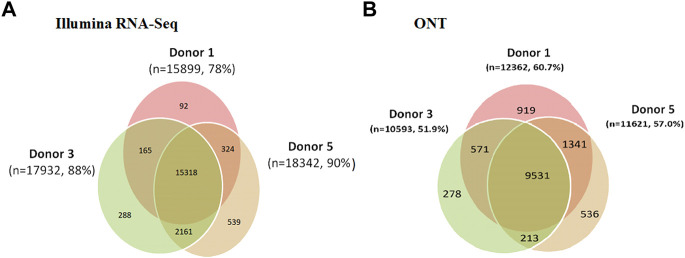
Results of the analysis of the three human liver tissue samples (donor 1, donor 3, and donor 5) indicating all transcripts detected using **(A)** Illumina RNA-Seq data (FPKM > 0) and **(B)** ONT (TPM > 0).

Comparison of the variability in the coverage of expressed genes in the transcriptome profiling results obtained using the Illumina RNA-Seq and ONT methods is shown in Figure 6. The results obtained for Chr18 are also typical of the entire genome. The greatest coverage was achieved using Illumina RNA-Seq technology. The proportion of transcripts detected simultaneously in all three donor samples was approximately 70% of the total number of transcripts detected using both Illumina RNA-Seq and ONT. When comparing the results of the transcriptome profiling of liver tissues of the three donors using Illumina RNA-Seq technology, it can be seen that the largest number of genes detected in only one donor was found in the liver of donor #5. In the case of ONT, the most number of genes was detected in only one donor (donor #1). The number of genes detected only in donor #5 using ONT was 11,639.

When comparing the results obtained using next-generation sequencing of the Chr18 transcriptome using the three platforms for each donor, the following observations were made. The intersection of the results from the three platforms was 49.8% (137 transcripts were detected in donor #3), 59% (163 transcripts for donor #1), and 56% (154 transcripts were detected in donor #5; see Figure 5) of Chr18 genes. The highest coverage of the Chr18-centric transcriptome for each donor was achieved using Illumina RNA-Seq.

A similar pattern was observed in the transcriptome profiling of liver tissues from the three donors. Because PCR is a targeted technique, its application to the entire genome is time-consuming and expensive; therefore, when studying variability in the coverage of expressed genes throughout the genome, we used only two technologies: Illumina RNA-Seq and ONT.

Comparison of the variability in the coverage of expressed genes in the transcriptome profiling results obtained using the Illumina RNA-Seq and ONT methods is shown in Figure 6. The results obtained for Chr18 are also typical of the entire genome. The greatest coverage was achieved using Illumina RNA-Seq technology. The proportion of transcripts detected simultaneously in all three donor samples was approximately 70% of the total number of transcripts detected using both Illumina RNA-Seq and ONT. When comparing the results of transcriptome profiling of liver tissues of the three donors using Illumina RNA-Seq technology, it can be seen that the largest number of genes detected in only one donor was found in the liver of donor #5. In the case of ONT, the most number of genes was detected in only one donor (donor #1). The number of genes detected only in donor #5 using ONT was 11,639.

## Discussion

Table 1 presents the results of the genome coverage comparison of each human chromosome using either ONT or Illumina RNA-Seq (HiSeq) technology. It can be observed that our scheme provides stable results for all human chromosomes. In other words, when using a cut-off level>0, most coverage was observed for Illumina-derived data. The application of ONT does not provide a significant increase in gene-centric coverage at the genome level; therefore, the application of this technology for panoramic studies of the transcriptome of a single chromosome or the entire genome is not optimal (at least in the format of direct RNA sequencing used). It has been shown that Illumina allows for 89%–97% chromosome genome coverage, while the total coverage of the chromosome genome using ONT and Illumina equally ranges from 90% to 99%. ONT alone provides 30%–72% coverage; therefore, it cannot be used for full genome profiling.

**TABLE 1:** Distribution of transcripts detected in the liver tissue samples by the Illumina RNA-Seq (FPKM > 0) and ONT (TPM > 0) methods across human chromosomes.

Chr	Number of protein-coding genes	Number of detected transcripts (Illumina RNA-Seq)	Number of detected transcripts (ONT)	Number of transcripts in set intersections (Illumina RNA-Seq and ONT)	Proportion of transcripts detected using Illumina RNA-Seq	Proportion of transcripts detected using ONT	Total
1	2,022	1,951	1,361	1,330	0.96	0.67	0.98
2	1,247	1,210	895	881	0.97	0.71	0.98
3	1,059	1,002	762	727	0.94	0.71	0.97
4	755	726	507	492	0.96	0.67	0.98
5	856	832	591	580	0.97	0.69	0.98
6	972	936	674	654	0.96	0.69	0.98
7	962	924	621	611	0.96	0.64	0.97
8	675	659	451	444	0.97	0.66	0.98
9	778	760	547	539	0.97	0.70	0.98
10	720	699	509	500	0.97	0.70	0.98
11	1,281	1,249	777	760	0.97	0.60	0.98
12	1,013	996	708	699	0.98	0.69	0.99
13	326	315	226	218	0.96	0.69	0.99
14	662	643	447	443	0.97	0.67	0.97
15	589	581	399	395	0.98	0.67	0.99
16	822	802	598	590	0.97	0.72	0.98
17	1,126	1,100	766	751	0.97	0.68	0.99
18	275	267	186	185	0.97	0.67	0.97
19	1,396	1,342	949	932	0.96	0.67	0.97
20	537	508	355	344	0.94	0.66	0.96
21	209	201	137	132	0.96	0.65	0.98
22	459	442	317	309	0.96	0.69	0.98
X	812	729	453	430	0.89	0.55	0.92
Y	40	37	12	11	0.92	0.03	0.95
**Total/average**	**19,593**	**18,911**	**13,248**	**12,957**	**0.96**	**0.65**	**0.98**

Sex chromosomes evidently differ in the parameter “the proportion of transcripts detected using the ONT method.” Probably, the poor coverage is due to the fact that short fragments of transcripts cannot be detected by nanopore sequencing. The values of this parameter are only 55% and 3% for the X and Y chromosomes, respectively (Table 1), which are significantly lower than those for somatic chromosomes.

Thus, the method of choice for transcriptomic analysis that achieves the maximum coverage for which at least one transcript has been detected (maximum chromosome-centric coverage) was found to be Illumina RNA-Seq (FPKM > 0).

The underlying reason for the observed differences may be a much higher expression of genes in actively proliferating HepG2 cells at average, alongside qualitatively distinct expression profiles for HepG2 cells and liver hepatocytes, as earlier revealed by the qPCR analysis of the human chromosome 18 transcripts [in terms of transcript abundance in the number of copies per cell (Kiseleva et al., 2018)]. More than half of chromosome 18 genes are expressed higher in HepG2 cells than in hepatocytes (by a factor of 4 or above). Moreover, the expression profile for chromosome 18 genes was found to be bimodal in the case of HepG2 cells and unimodal for liver tissue. Altogether, it may result in the effective detection of highly and moderately expressed genes encoded on chromosome 18 by both Illumina and ONT, thus providing the close coverage of expressed genes, varying mostly due to technical reasons related to the use of different technological platforms. Consequently, the two technologies complement each other in regard to the coverage of expressed genes in the case of HepG2 cells. Bioinformatics algorithms, number, and the length of reads also can influence the results. In this study, we aimed to estimate the influence of the TPM cut-off level, but in our future research studies, we aim to estimate the impact of other aforementioned parameters.

## Materials and methods

### Human liver samples

Human liver samples were collected by autopsy from three male donors, hereafter referred to as donors #1, #3, and #5 aged 65, 38, and 54 years, respectively. The donors tested negative for HIV and hepatitis, and the sections showed no histological signs of liver disease. The postmortem resected samples were immediately placed in RNAlater Stabilization Solution (Thermo Fisher Scientific, United States) and stored at –20°C until further use.

### Data

The results of transcriptome profiling using the three technologies (qPCR, Illumina RNA-Seq, and ONT) for chromosome 18-encoded genes in the liver tissue obtained by the Russian Human Proteome Consortium were analyzed. Detailed information about the samples, sample preparation, and experimental procedure is described by Krasnov et al. (2021). Our study dealt only with RNA-related data. Here, we worked on the datasets that were previously published in the annual reports of the Russian Human Proteome Consortium (Zgoda et al., 2013; Ponomarenko et al., 2014; Poverennaya et al., 2016; Poverennaya et al., 2017). Illumina sequencing was performed using the Illumina HiSeq 2500 system (two lanes per eight samples). Nanopore sequencing was carried out using the MinION sequencer (ONT, United Kingdom) with FLO-MIN 106 flow cells and R9.4 chemistry and a direct RNA sequencing kit (SQK-RNA002, ONT, United Kingdom). The sequencing data are available in the NCBI Sequence Read Archive (BioProject ID: PRJNA635536). The description of experimental procedures also can be found in the Supplementary Material (S3: Experimental section).

### Bioinformatics analyses

Briefly, basecalling was performed by the Guppy basecalling (ONT) program, designed to translate ionic signals into a nucleotide sequence. The basecalling quality was controlled using the MinIONQC program, and values <7 were discarded. To align reads onto the genome, the Minimap2 program was used with the option -ax splice and -junc-bed to account for splicing (the junc-bed option enables alignment with long gapes, indicating splicing sites). Using the Salmon quant subprogram, the content of the transcripts in the samples was calculated, expressed as the number of reads, and also in normalized TPM units. Protein-coding genes were selected from the resulting files using the GENCODE 38 genome assembly (GRCh40 release).

Illumina FASTQ files were processed by Trimmomatic within options paired-ends and minimal length of read equal to 100 base pairs. After that, quality was controlled by FastQC and reads of phread >30 were quantified by the Salmon quant command and expressed in FPKM units.

The Tanimoto index (T) was used to compare the sets of transcripts obtained using different methods (Rogers and Tanimoto, 1960). The Tanimoto index measures the proximity of non-quantitative datasets (Bajusz et al., 2015). The coefficient of similarity T (*a*, *b*) between two sets *a* and *b* was calculated as follows (Rogers and Tanimoto, 1960):

$$T\left(a,\;b\right)=\left|Pab\right|/\left(\left|Pa\right|+\left|Pb\right|-\left|Pab\right|\right),$$ (1)

where *Pa* indicates the variety of set *a*, *Pb* indicates the variety of set *b*, and *Pab* indicates the variety of transcripts shared between sets *a* and *b*.

If the Tanimoto index is within 1.0–0.7, it is considered that the two sets are identical; Tanimoto index values from 0.75 to 0.55 indicate that the similarity is much weaker, and values of 0.55 and below indicate that the arrays differ considerably (Ilgisonis et al., 2021). Quantitative data (FPKM, TPM, and the number of transcripts per cell estimated by qPCR) were compared when creating a calculation model for converting FPKM (TPM) data into the number of transcripts per cell. Calibration curves were constructed using qPCR data for the results obtained using Illumina RNA-Seq and ONT. The determination coefficient (*R* ^2^) was used to assess the relationship between the quantitative datasets.

## Conclusion

In this study, we have shown that the variability in the coverage of expressed genes in the results of transcriptome profiling of liver tissues of three donors using three technologies, Illumina RNA-Seq, qPCR, and ONT, was no more than 5%. It has been shown that the application of Illumina RNA-Seq technology (when FPKM > 0) allowed for obtaining the maximum transcriptome coverage in human liver tissue samples by detecting at least one transcript corresponding to each of the approximately 96% protein-coding genes in the human genome, which is true for the gene sets located on each human chromosome. For comparison, ONT (TPM > 0) covered only 65% of the transcriptome for the same samples. Therefore, for analyzing the HepG2 cell line, the most complete coverage of the transcriptome was provided by a combination of two technologies (Illumina RNA-Seq and ONT), whereas for achieving the maximum coverage of the liver tissue transcriptome, a single technology, Illumina RNA-Seq, was used.

## Data Availability

The data presented in the study are deposited in the NCBI Sequence Read Archive, BioProject ID: PRJNA635536.

## Supplementary material

SUPPLEMENTARY MATERIAL S1 Venn diagrams of the chromosomes (coverage by Illumina RNA-Seq and ONT).

SUPPLEMENTARY MATERIAL S2 Initial data: excel table—by donors and average values.

SUPPLEMENTARY MATERIAL S3 Experimental section.

SUPPLEMENTARY MATERIAL S4 Raw data.
