# Supplementary material for: Gene-centric coverage of the human liver transcriptome: QPCR, Illumina, and Oxford Nanopore RNA-Seq
Source: Front Mol Biosci. 2022 Dec 5;9:944639. doi: 10.3389/fmolb.2022.944639 (PMC9760921; doi:10.3389/fmolb.2022.944639)
Supplement: Supplementary file 2 [file DataSheet3.docx]

**SUPPLEMENTARY MATERIAL**

**Gene-Centric Coverage jf Human Liver Transcriptome: qPCR, Illumina and Oxford Nanopore RNA-seq**

**Ekaterina V. Ilgisonis, Elena A. Ponomarenko, Svetlana N. Tarbeeva, Andrey V. Lisitsa, Victor G. Zgoda, Sergey P. Radko, Alexander I. Archakov**

Institute of Biomedical Chemistry, Moscow, Russia, 119121

**Experimental Section**

**Human liver samples and HepG2 cells**

Samples of human liver were collected at autopsy from 3 male donors (designated further as donors 1, 3, and 5) aged 65, 38, and 54 years with the approval of the N.I. Pirogov Russian State Medical University Ethical Committee (protocol #3; March 15, 2018) with the informed consent from donor’s representatives. The donors were HIV and hepatitis free, and the sections had no histological signs of liver diseases. The postmortem resected samples were immediately placed into RNAlater RNA Stabilization Solution (Thermo Fisher Scientific, USA) and stored at -20°C until further use.

**Transcriptome profiling using reverse transcription qPCR**

For transcriptome profiling with qPCR, total RNA was isolated from liver tissue samples with the RNeasy Mini Kit (Qiagen, Germany) according to the manufacturer’s protocol. The on-column DNase digestion step was performed using the RNаse-Free DNase Set (Qiagen, Germany). The isolated total RNA was quantified on a Qubit 4 fluorometer, employing the Qubit RNA HS Assay Kit (Thermo Fisher Scientific, USA), and the RNA quality was assessed on a Bioanalyzer 2100 System (Agilent Technologies, USA). The RIN numbers for all preparations of total RNA were 7.5 or higher. Synthesis of cDNA was carried out using the AffinityScript qPCR cDNA Synthesis Kit and random primers (Agilent Technologies, USA) according to the manufacturer’s recommendations. The cDNA samples were stored at -20°C until further use.

The amount of each transcript encoded on Chr18 was assessed by measuring the number of copies of pertinent cDNA in the cDNA preparation derived from total RNA. qPCR was conducted in two formats—droplet digital PCR (ddPCR; 49 transcripts) and PCR in real time (226 transcripts), employing the earlier designed set of primers and probes [1, 2], with minor exceptions. While ddPCR was performed as described previously [1, 2], the transcriptome profiling by real time PCR was carried out using the Δ*Ct* method [3].

To calculate the copy number of a transcript per cell, the copy number per PCR probe was normalized by dividing it by the amount of total RNA in the PCR probe (200 ng). The number of transcripts per nanogram of total RNA was brought to the copy numbers per cell based on the amount of total RNA in hepatocytes and HepG2 cells, reported to equal 40 pg/cell [4].

**Illumina HiSeq sequencing and bioinformatics analysis**

Total RNA was isolated using the Extract RNA kit (Eurogen, Russia). RNA quality was evaluated using the Bioanalyzer 2100 System (Agilent Technologies, USA). The RIN numbers varied from 7.3 to 9.1. Clustering and sequencing were carried out using the Illumina HiSeq 2500 system (2 lanes per 8 samples) according to the manufacturer’s protocols (Denature and Dilute Libraries Guide; Sequencing in Rapid Run Mode). For each replicate, we derived from 32 to 59 million reads.

The derived fastq files were analyzed by FastQC and then were processed by Trimmomatic. The read mapping and expression quantification were carried out employing STAR 2.7 (splice-aware mapping to genome), bowtie2 (mapping to transcripts), RSEM 1.3 (quantifications of the reads), and Salmon (quasi-mapping and quantification) software packages. The genome GRCh38.p12 assembly (Ensembl release 97) was used as a reference. Finally, we compared the results obtained with STAR-RSEM, bowtie2-RSEM and Salmon, calculated the Spearman/Pearson correlation coefficients and created clustering dendrograms. The distance between samples/pipelines (i.e., dissimilarity rate) was evaluated as 1–corr.coeff. To create dendrograms, we used the complete linkage hierarchical clustering method.

The sequencing data obtained in this study is available at NCBI Sequence Read Archive (BioProject ID PRJNA635536).

**MinION sequencing and bioinformatics analysis**

Total RNA was isolated and characterized as that for the qPCR analysis (seeabove). The extraction of mRNA from the total RNA preparations was conducted using the Dynabeads™ mRNA Purification Kit (Thermo Fisher Scientific) following the manufacturer’s recommendations. The mRNA preparations were immediately frozen and stored at -80°C until nanopore sequencing.

Nanopore sequencing was carried out using the MinION sequencer (Oxford Nanopore Technologies (ONT), UK) with FLO-MIN106 flow cells and R9.4 chemistry and the Direct RNA sequencing kit (SQK-RNA002, ONT). The sequencing libraries were prepared following the manufacturer’s protocol. The SuperScript III Reverse Transcriptase (Thermo Fisher Scientific) and NEBNext Quick Ligation Module (New England Laboratories, USA) were used for reverse transcription and for end repair and ligation, respectively. The Agencourt RNAClean XP magnetic beads (Beckman Coulter, USA) were employed for nucleic acid purification. The libraries were sequenced in 48-h runs. The overall outputs and median lengths of reads are provided in the table below (Table S).

Table S. The characteristics of MinION sequencing for liver samples.

| Sequencing characteristics | Donors | | |
| --- | --- | --- | --- |
|  | 1 | 3 | 5 |
| The overall yield, Gb | 1.22 | 0.51 | 0.57 |
| The number of reads, mln | 1.44 | 0.66 | 0.99 |
| Median length of reads, kb | 1.37 | 1.02 | 0.84 |

The fast5 files produced by MinION were uploaded onto the Amazon Web Services ElasticCloud2 and processed using the GPU-powered (Nvidia Tesla V100) virtual instance p3.2xlarge (8x2.7 GHz vCPUs, 1 GPU) by the ONT-provided basecalling software guppy_basecaller [5] with parameters “-flowcell FLO-MIN107 -kit SQK-RNA002”. Further pipelines included the quality control by the MinIONqc.R script, followed by mapping the reads onto the gencode.v32.transcriptome using minimap2 v. 2.17 [6]. The overall statistics of alignment mapping was produced using the “samtools stats” command, and the quantitative data were further collected by executing the program Salmon. 0.12/1.1.0 with the command line options “quant -p 8 –noErrorModel” [7].

REFERENCES

1. Ponomarenko, E. A.; Kopylov, A. T.; Lisitsa, A. V.; Radko, S. P.; Kiseleva, Y. Y.; Kurbatov, L. K.; Ptitsyn, K. G.; Tikhonova, O. V.; Moisa, A. A.; Novikova, S. E.; Poverennaya, E. V.; Ilgisonis, E. V.; Filimonov, A. D.; Bogolubova, N. A.; Averchuk, V. V.; Karalkin, P. A.; Vakhrushev, I. V.; Yarygin, K. N.; Moshkovskii, S. A.; Zgoda, V. G.; Sokolov, A. S.; Mazur, A. M.; Prokhortchouck, E. B.; Skryabin, K. G.; Ilina, E. N.; Kostrjukova, E. S.; Alexeev, D. G.; Tyakht, A. V.; Gorbachev, A. Y.; Govorun, V. M.; Archakov, A. I. Chromosome 18 Transcriptoproteome of Liver Tissue and HepG2 Cells and Targeted Proteome Mapping in Depleted Plasma: Update 2013. J. Proteome Res. 2014, 13 (1), 183–190. <https://doi.org/10.1021/pr400883x>.

2. Poverennaya, E. V.; Kopylov, A. T.; Ponomarenko, E. A.; Ilgisonis, E. V.; Zgoda, V. G.; Tikhonova, O. V.; Novikova, S. E.; Farafonova, T. E.; Kiseleva, Y. Y.; Radko, S. P.; Vakhrushev, I. V.; Yarygin, K. N.; Moshkovskii, S. A.; Kiseleva, O. I.; Lisitsa, A. V.; Sokolov, A. S.; Mazur, A. M.; Prokhortchouk, E. B.; Skryabin, K. G.; Kostrjukova, E. S.; Tyakht, A. V.; Gorbachev, A. Y.; Ilina, E. N.; Govorun, V. M.; Archakov, A. I. State of the Art of Chromosome 18-Centric HPP in 2016: Transcriptome and Proteome Profiling of Liver Tissue and HepG2 Cells. J. Proteome Res. 2016, 15 (11), 4030–4038. <https://doi.org/10.1021/acs.jproteome.6b00380>.

3. Riedel, G.; Rüdrich, U.; Fekete-Drimusz, N.; Manns, M. P.; Vondran, F. W. R.; Bock, M. An Extended ΔCT-Method Facilitating Normalisation with Multiple Reference Genes Suited for Quantitative RT-PCR Analyses of Human Hepatocyte-like Cells. PLoS One 2014, 9 (3). <https://doi.org/10.1371/journal.pone.0093031>.

4. Wilkening, S.; Stahl, F.; Bader, A. Comparison of Primary Human Hepatocytes and Hepatoma Cell Line HepG2 with Regard to Their Biotransformation Properties. Drug Metab. Dispos. 2003, 31 (8), 1035–1042. <https://doi.org/10.1124/dmd.31.8.1035>.

5. Wick, R. R.; Judd, L. M.; Holt, K. E. Performance of Neural Network Basecalling Tools for Oxford Nanopore Sequencing. Genome Biol. 2019, 20 (1). <https://doi.org/10.1186/s13059-019-1727-y>.

6. Li, H. Minimap2: Pairwise Alignment for Nucleotide Sequences. Bioinformatics 2018, 34 (18), 3094–3100. <https://doi.org/10.1093/bioinformatics/bty191>.

7. Patro, R.; Duggal, G.; Love, M. I.; Irizarry, R. A.; Kingsford, C. Salmon Provides Fast and Bias-Aware Quantification of Transcript Expression. Nat. Methods 2017, 14 (4), 417–419. https://doi.org/10.1038/nmeth.4197.
