# Supplementary material for: Gene-centric coverage of the human liver transcriptome: QPCR, Illumina, and Oxford Nanopore RNA-Seq
Source: Front Mol Biosci. 2022 Dec 5;9:944639. doi: 10.3389/fmolb.2022.944639 (PMC9760921; doi:10.3389/fmolb.2022.944639)

chromosome 1
protein-coding genes: 2022
detected transcripts: 2016 (98%)

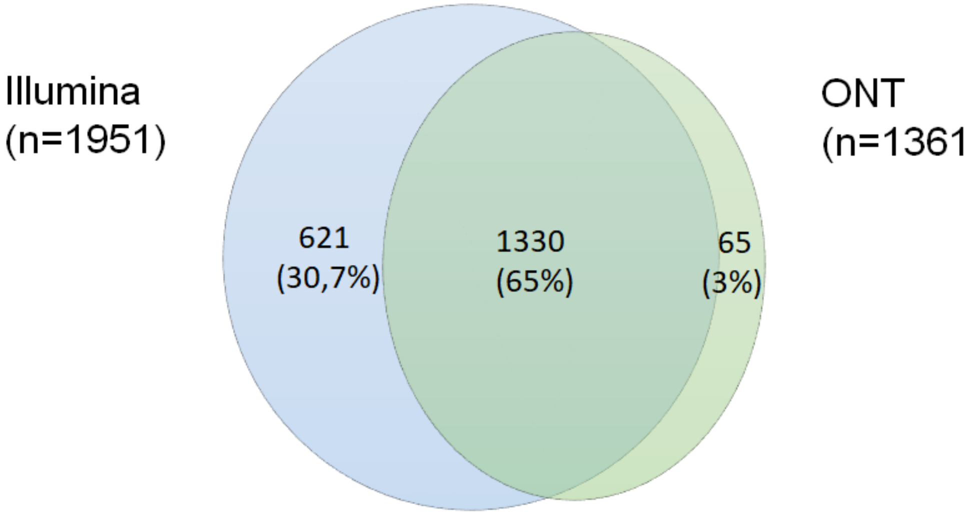

chromosome 2
protein-coding genes: 1247
detected transcripts: 1224 (98%)

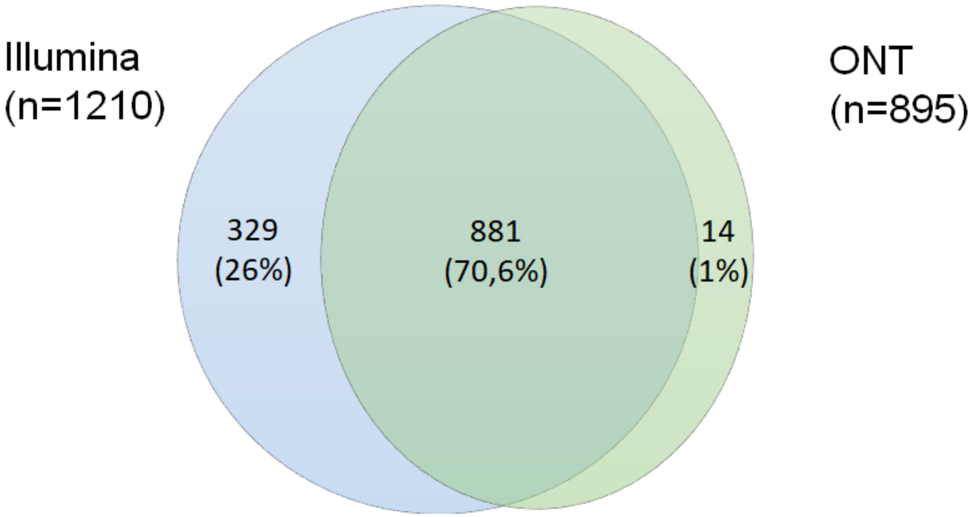

chromosome 3
protein-coding genes: 1059
detected transcripts: 1037 (98%)

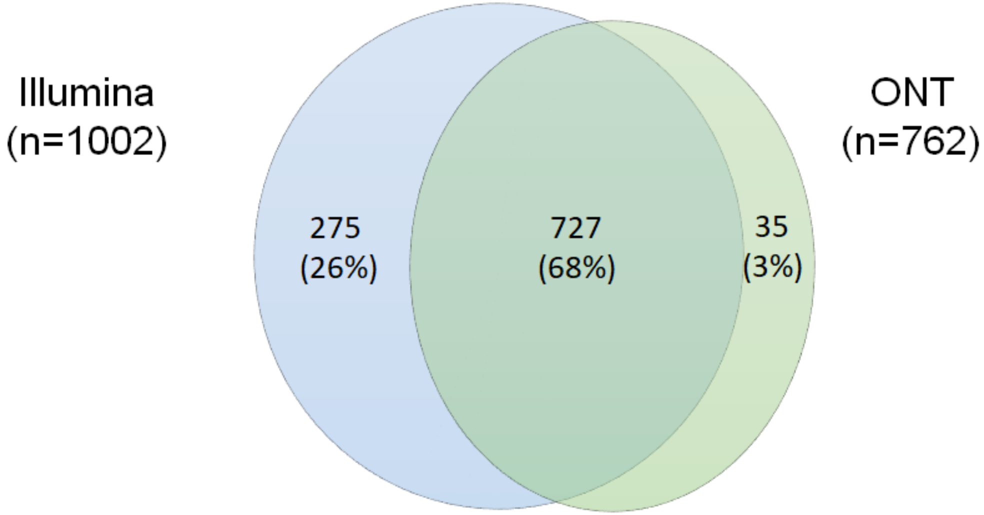

chromosome 4
protein-coding genes: 775
detected transcripts: 741 (96%)

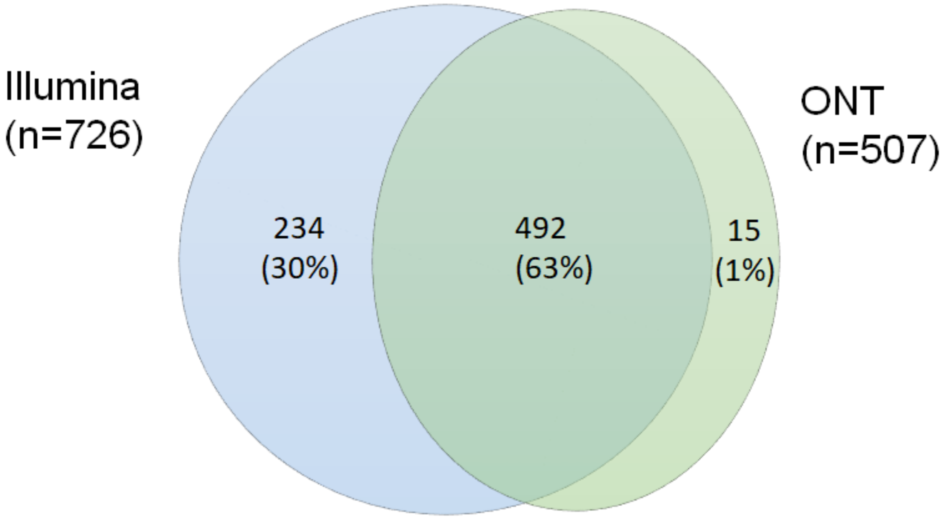

chromosome 5
protein-coding genes: 856
detected transcripts: 843 (98%)

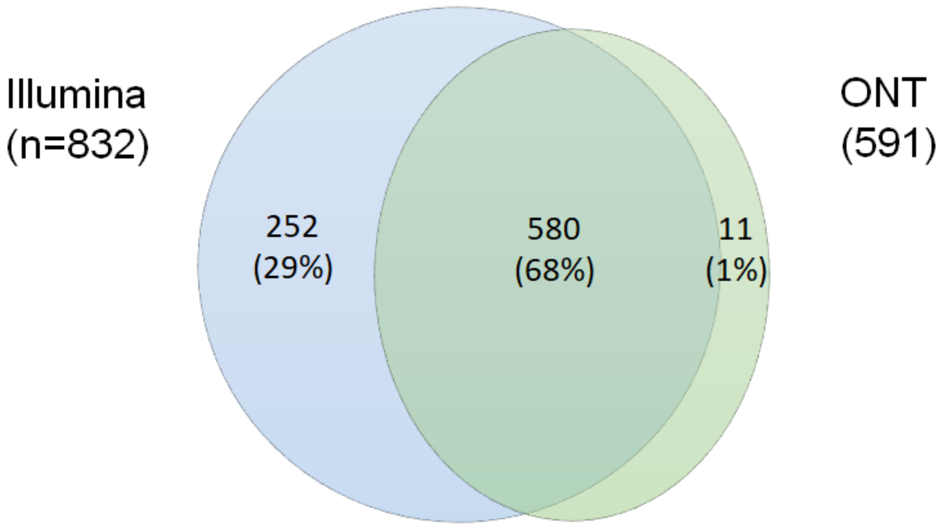

chromosome 6
protein-coding genes: 972
detected transcripts: 956 (98%)

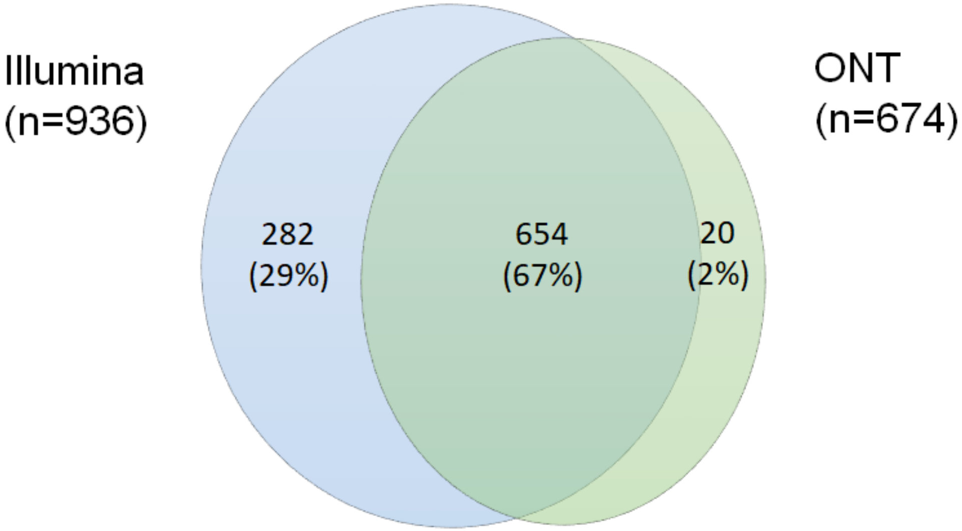

chromosome 7
protein-coding genes: 962
detected transcripts: 934 (97%)

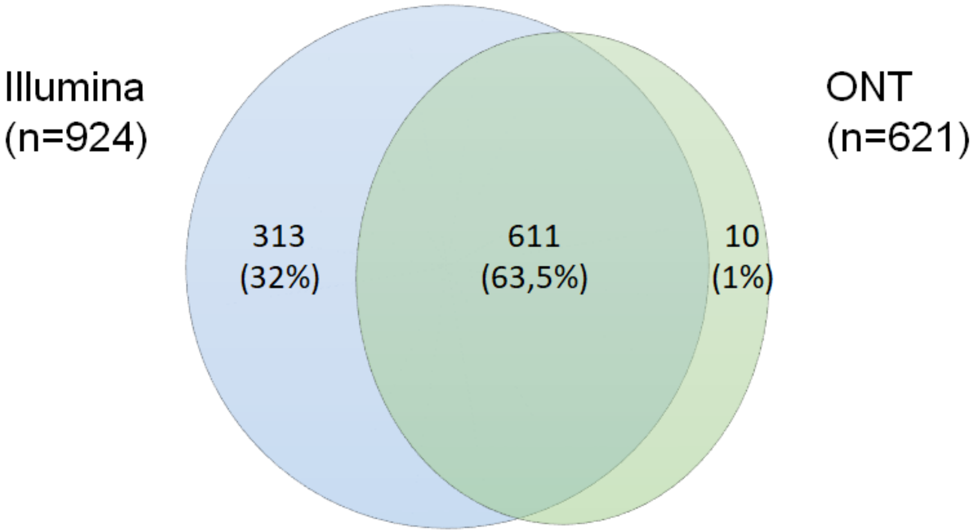

chromosome 8
protein-coding genes: 675
detected transcripts: 666 (98%)

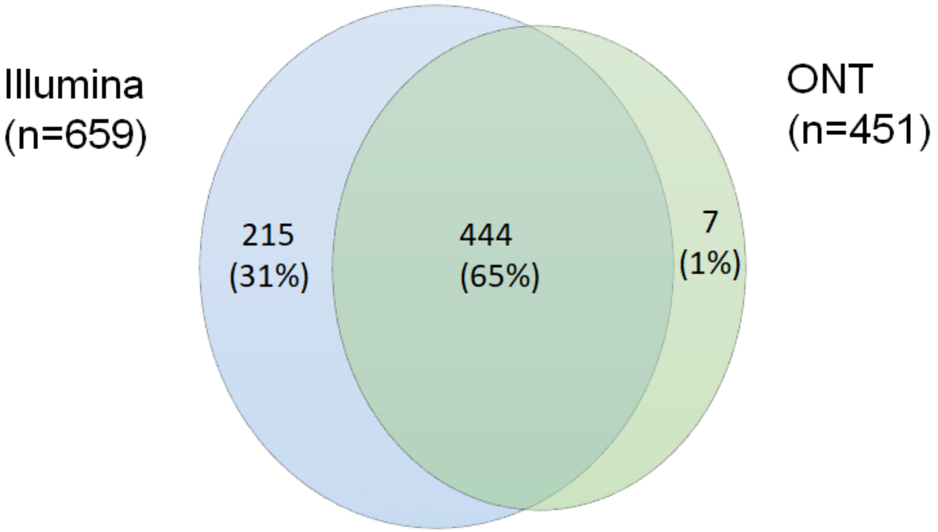

chromosome 9
protein-coding genes: 778
detected transcripts: 768 (98%)

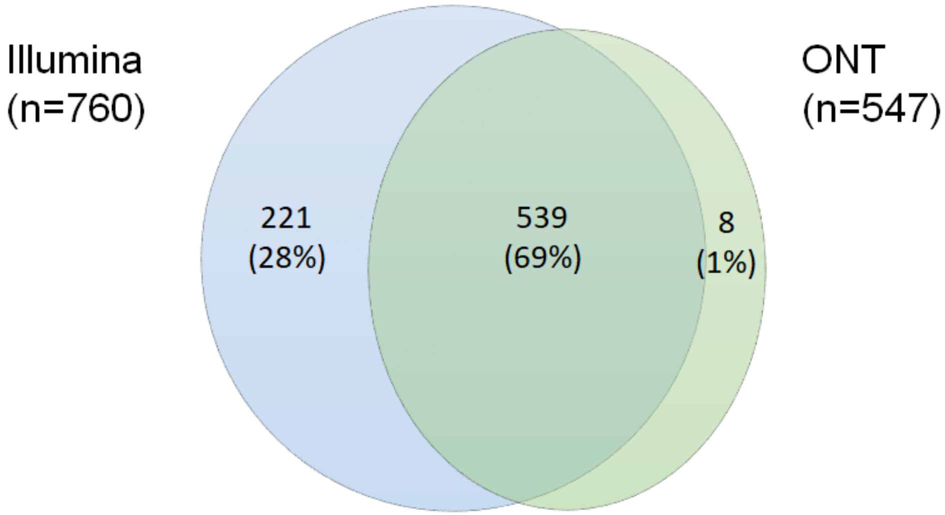

chromosome 10
protein-coding genes: 720
detected transcripts: 708 (98%)

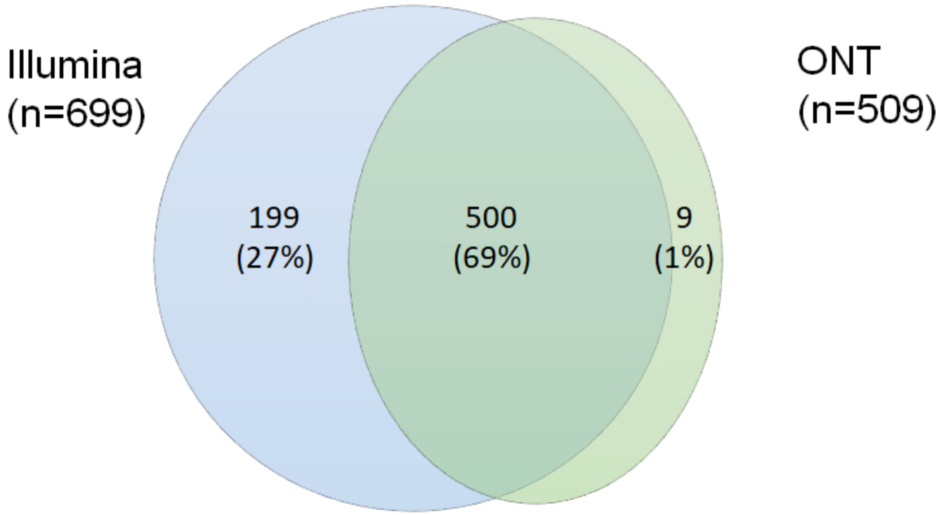

chromosome 11
protein-coding genes: 1281
detected transcripts: 1266 (98%)

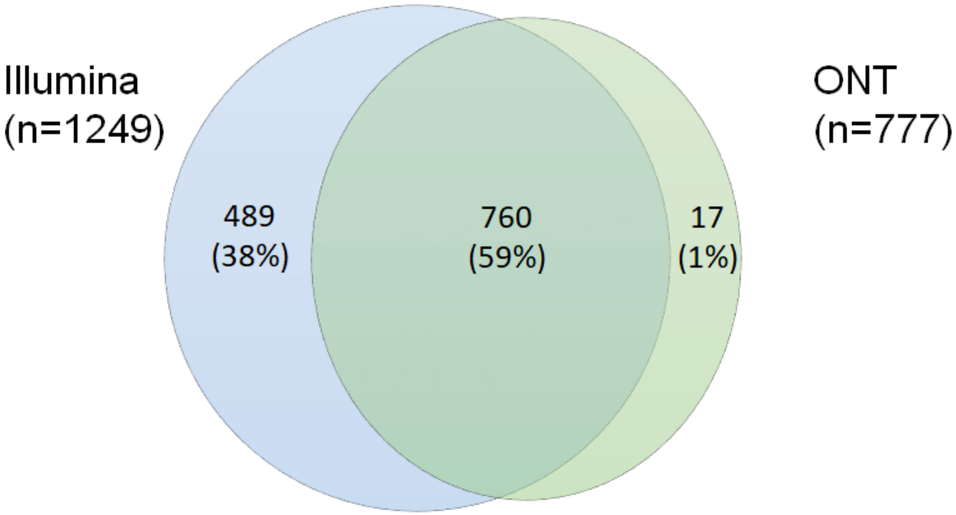

chromosome 12
protein-coding genes: 1013
detected transcripts: 1005 (98%)

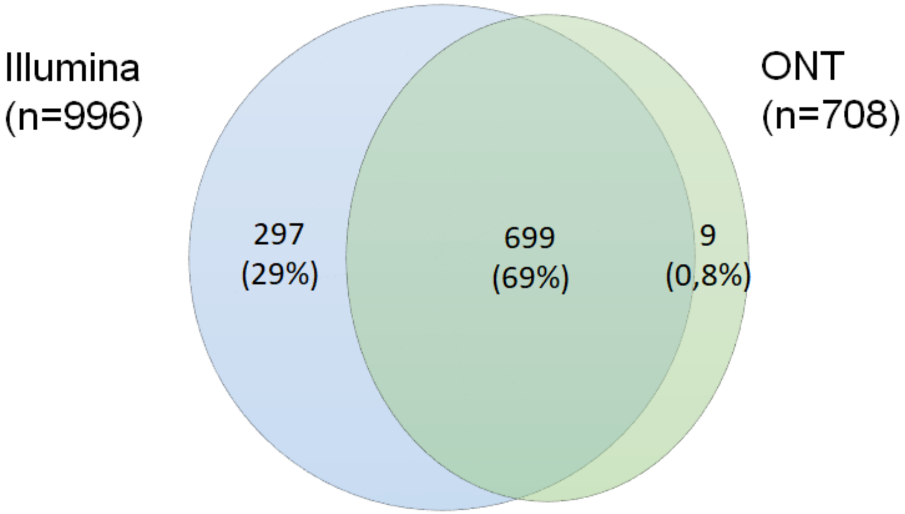

chromosome 13
protein-coding genes: 326
detected transcripts: 323 (99%)

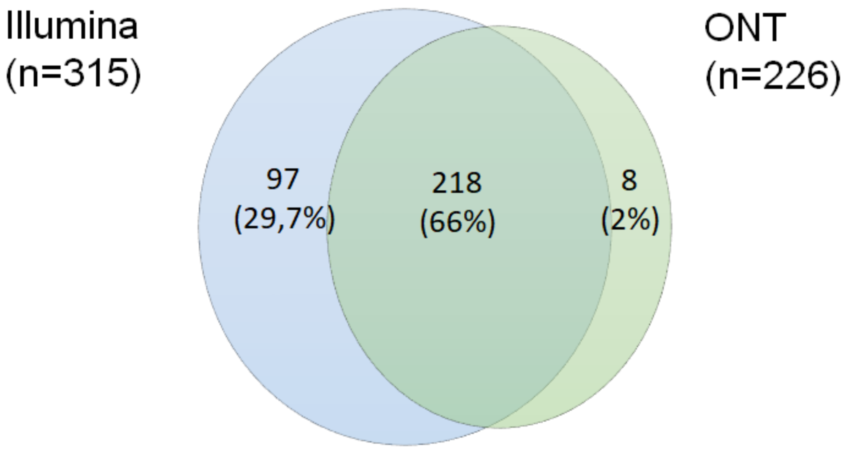

chromosome 14
protein-coding genes: 662
detected transcripts: 647 (98%)

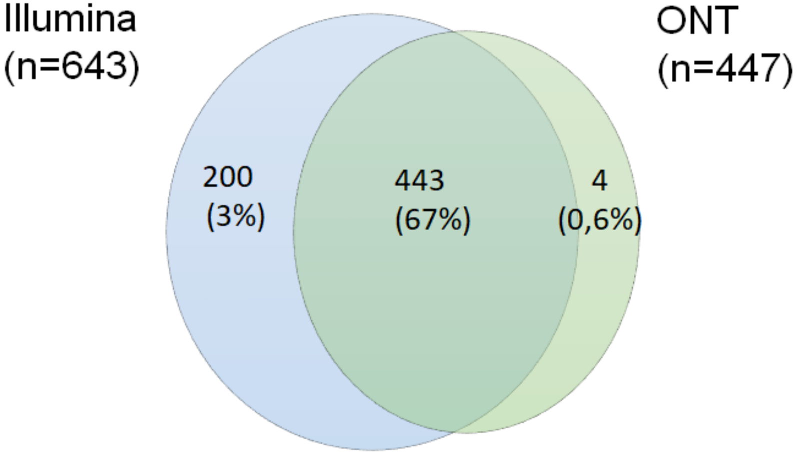

chromosome 15
protein-coding genes: 589
detected transcripts: 585 (99%)

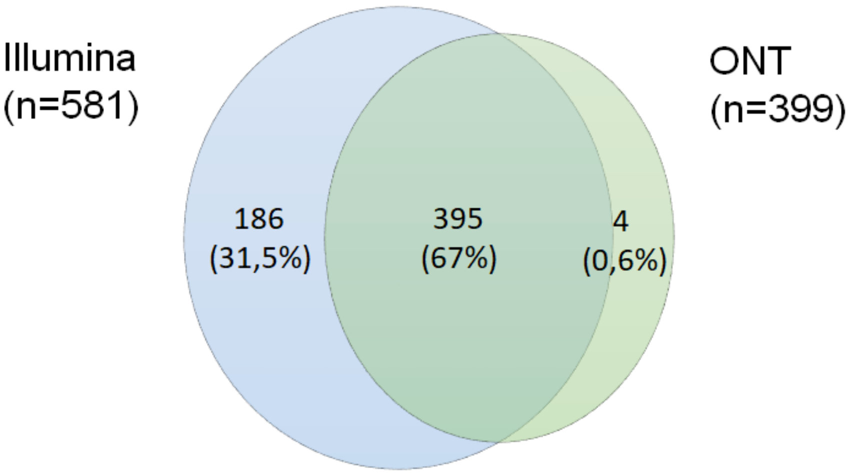

chromosome 16
protein-coding genes: 822
detected transcripts: 810 (98%)

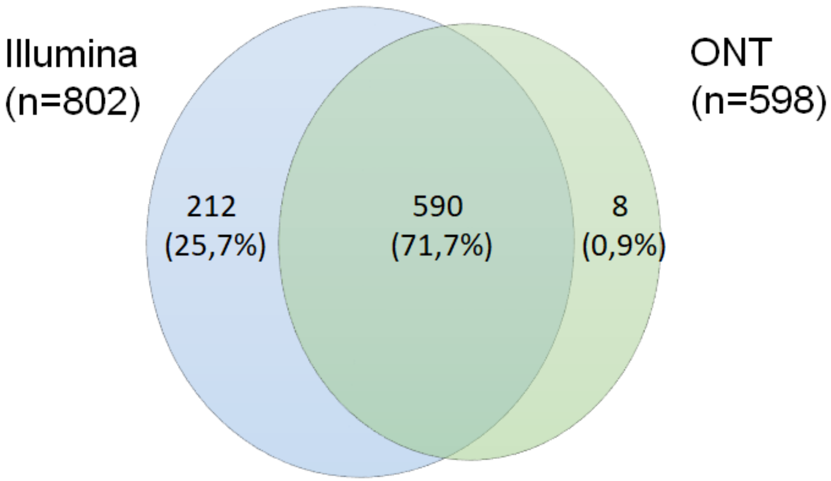

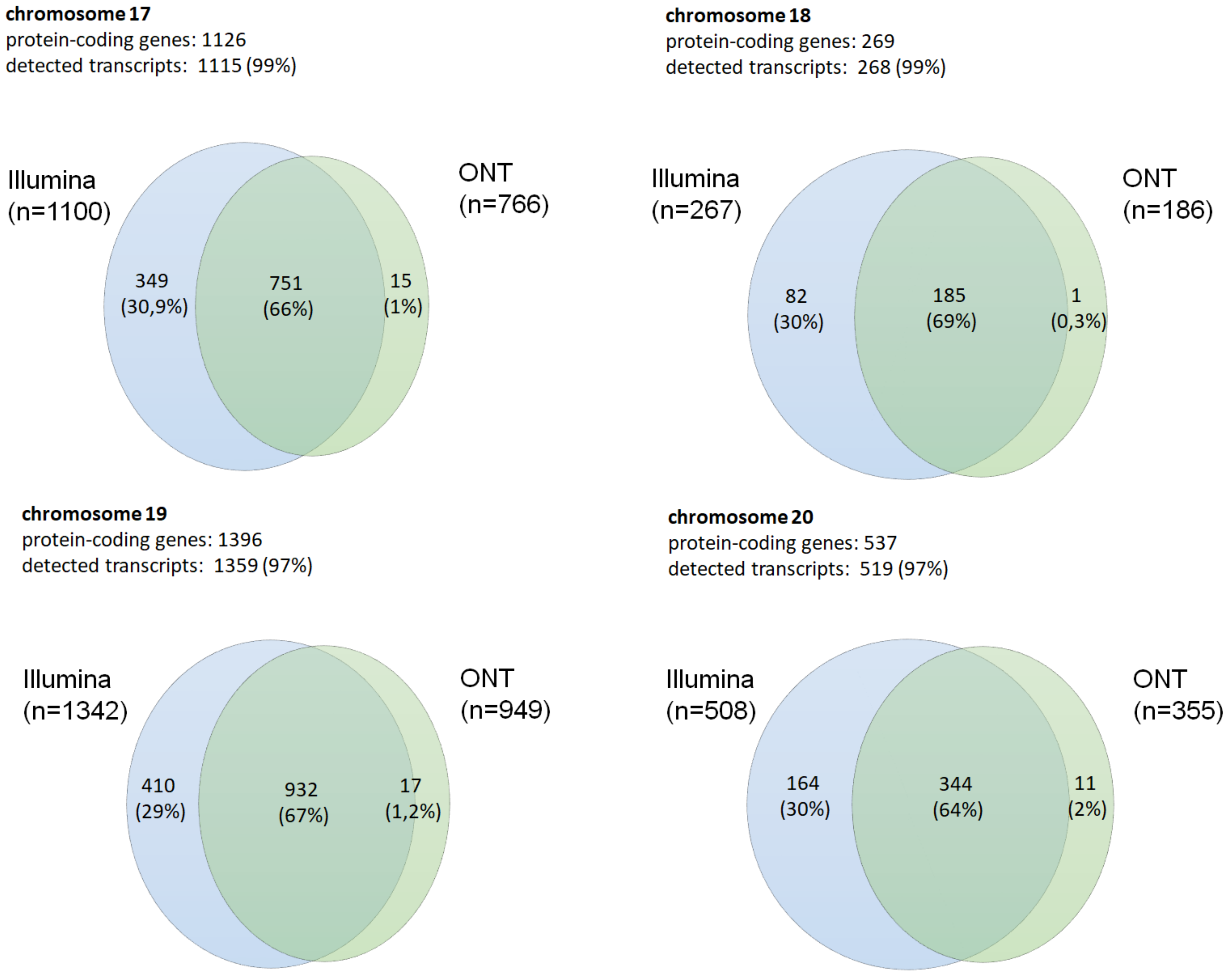
chromosome 17
protein-coding genes: 1126
detected transcripts: 1115 (99%)
Illumina (n=1100)
ONT (n=766)
349 (30,9%)
751 (66%)
15 (1%)
chromosome 18
protein-coding genes: 269
detected transcripts: 268 (99%)
Illumina (n=267)
ONT (n=186)
82 (30%)
185 (69%)
1 (0,3%)
chromosome 19
protein-coding genes: 1396
detected transcripts: 1359 (97%)
Illumina (n=1342)
ONT (n=949)
410 (29%)
932 (67%)
17 (1,2%)
chromosome 20
protein-coding genes: 537
detected transcripts: 519 (97%)
Illumina (n=508)
ONT (n=355)
164 (30%)
344 (64%)
11 (2%)

chromosome 21
protein-coding genes: 209
detected transcripts: 519 (98%)

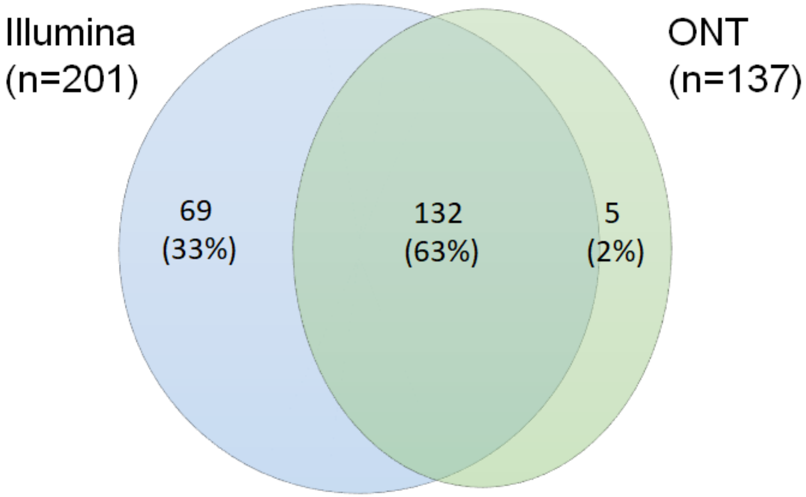

chromosome 22
protein-coding genes: 459
detected transcripts: 450 (98%)

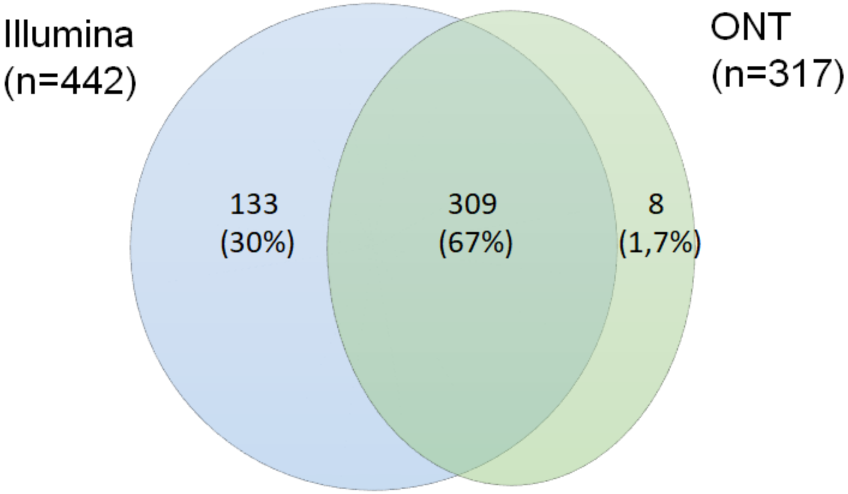

chromosome X
protein-coding genes: 812
detected transcripts: 752 (92,6%)

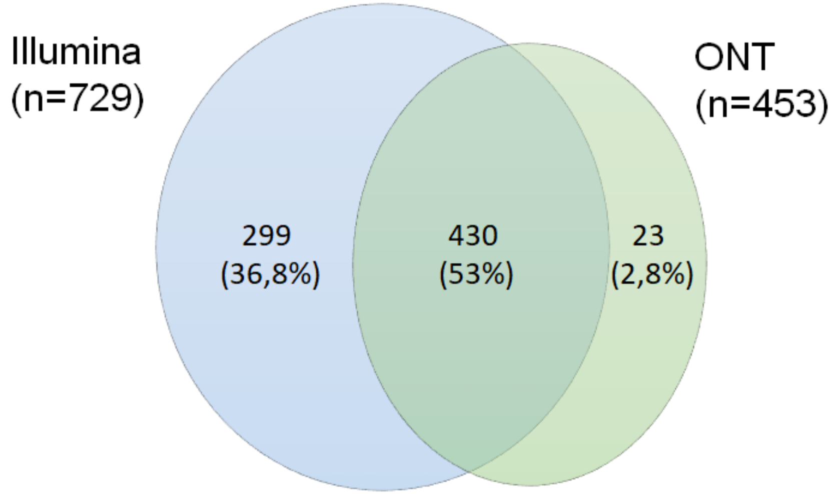

chromosome Y
protein-coding genes: 40
detected transcripts: 38 (94,5%)

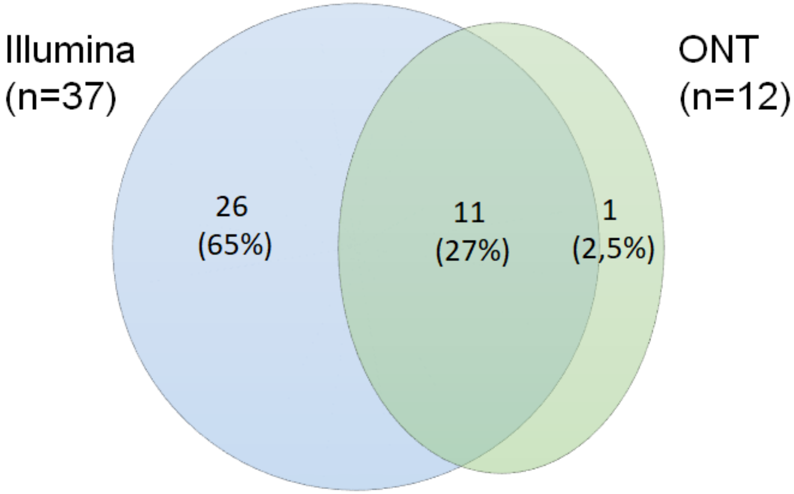

Mitochondrial DNA
protein-coding genes: 16
detected transcripts: 15 (94%)

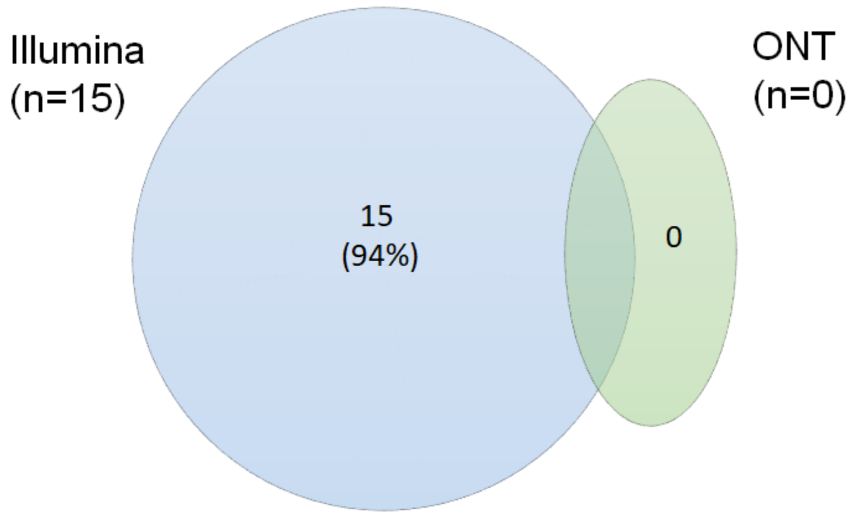

Supplement: Supplementary file 3 [file DataSheet1.PDF]
